# Biomarker validity in the critically ill: all must face the (continuous) renal replacement challenge!

**DOI:** 10.1186/s13054-015-1139-7

**Published:** 2015-12-06

**Authors:** Patrick M. Honore, Rita Jacobs, Inne Hendrickx, Elisabeth De Waele, Viola Van Gorp, Herbert D. Spapen

**Affiliations:** ICU Department, Universitair Ziekenhuis Brussel, Vrije Universiteit Brussel, 101 Laarbeeklaan, 1090 Jette Brussels, Belgium

We embrace with enthusiasm the recent study by Roderburg et al. [1] identifying osteopontin (OPN) as a valuable early prognostic biomarker in critically ill patients. Of note is that these investigators did not provide details on the incidence of acute kidney injury or the need for renal replacement therapy (RRT) in their patient population.

We recently argued that currently used markers of systemic inflammation such as C-reactive protein and procalcitonin are substantially cleared during continuous renal replacement therapy (CRRT) and thus may lose sensitivity to evaluate the degree and evolution of inflammation [2]. OPN is a highly negatively charged protein. Its nascent molecular weight (MW) approximates 32 kDa with slight variations due to post-translational modification or proteolytic cleavage [3]. OPN is not eliminated by slow extended dialysis [4]. This is not surprising, however, because the 5 kDa membrane cutoff point for molecular diffusion with this technique lies largely below the MW of OPN. Theoretically, continuous hemofiltration may remove OPN from the circulation but evidence is lacking. Moreover, CRRT is increasingly performed with novel membranes such as the surface-treated acrylonitrile 69 membrane [5]. Surface treatment implies coating with a polyethylene imine biopolymer resulting in a more neutral membrane surface composed of areas with a high density of positive charges. In addition to being highly biocompatible and permeable, this membrane displays potent adsorptive capacity.

More information regarding an eventual significant “loss” of OPN by filtration and/or membrane adsorption during CRRT is imperative before this protein can be accepted as a valid prognostic biomarker.

## Abbreviations

CRRT: continuous renal replacement therapy
MW: molecular weight
OPN: osteopontin
RRT: renal replacement therapy
