# Supplementary material for: Biomarker validity in the critically ill: all must face the (continuous) renal replacement challenge!
Source: Crit Care. 2015 Dec 6;19:426. doi: 10.1186/s13054-015-1139-7 (PMC4670724; doi:10.1186/s13054-015-1139-7)
Supplement: Related texts

# Sub article: Authors’ response: impact of renal replacement therapy on osteopontin levels in critically ill patients

**Authors:** Christoph Roderburg, Fabian Benz, Hans-Joerg Hippe, Tom Luedde, Christian Trautwein, Norbert Frey, Alexander Koch, Frank Tacke, Mark Luedde

We are grateful for the letter from Honore et al. raising the important question of an eventual “loss” of OPN by filtration during (C)RRT in critically ill patients. We cannot fully exclude this bias but we have performed additional analyses on our cohort of critically ill medical patients [1], suggesting that OPN serum levels at day 3 after admission to the ICU remain a valuable prognostic biomarker in critically ill patients. Although the records from our database only reveal whether patients received RRT or not (and, unfortunately, not the exact modalities, time points, and durations), OPN levels at day 3 were significantly higher in patients receiving RRT compared with patients without RRT (Fig. 1a), arguing against a significant loss due to filtration. In line with the prognostic role of OPN levels in critically ill patients, need for RRT (and thus presence of higher OPN levels) was associated with a dramatically impaired survival (Fig. 1b, c). Moreover, multivariate analysis including RRT as a variable revealed that OPN levels remained an independent prognostic marker in critically ill patients, as reported previously in our study [1]. Finally, OPN concentrations were strongly correlated to procalcitonin serum levels, which have been demonstrated to be independent of RRT in critical illness [6].

**Fig. 1:** OPN serum levels are elevated in critically ill patients with RRT. **a** OPN serum concentrations at day 3 of medical ICU treatment were significantly higher (*P* < 0.001, *U* test) in patients that received RRT (*n* = 34) compared with patients without need for this therapy (*n* = 78). **b**, **c** Patients with a need for RRT displayed a significantly impaired ICU survival and overall survival in comparison with other patients, based on Kaplan–Meier curve analysis. ***P <0.001. *RRT* Renal replacement therapy

Taken together, our results do not support the concern that OPN serum levels might not be a valid prognostic parameter in patients with acute kidney injury undergoing RRT. Nevertheless, we cordially thank Honore et al. for their valuable comment on our article, as further prospective trials considering renal failure and replacement should be conducted to properly address this question.
